# Prevalence and correlates of hypertension in Maharashtra, India: A multilevel analysis

**DOI:** 10.1371/journal.pone.0191948

**Published:** 2018-02-05

**Authors:** Mahadev D. Bhise, Shraboni Patra

**Affiliations:** Population Studies, International Institute for Population Sciences, Govandi Station Road, Deonar, Mumbai, Maharashtra, India; Pondicherry Institute of Medical Sciences, INDIA

## Abstract

**Background and aim:**

In the last few decades, the prevalence of hypertension has been drastically increased in India. The present study estimates the current prevalence of hypertension and its correlates in the state of Maharashtra. The variation in the prevalence of hypertension associated with individual-level characteristics is explained at the community and district level.

**Methods:**

Data is used from the recent round of District Level Household & Facility Survey (DLHS-4), 2012–13. The DLHS-4 has used the nationally representative sample, collected through multistage stratified sampling procedure. A similar sampling frame, used in NSSO-2007-08, has been followed. The chi-square test is used to show the significance level of the association between the estimated prevalence of hypertension and its correlates. Multilevel regression analysis is carried out to investigate the effects of individual and community level factors on the prevalence of hypertension.

**Results:**

The overall prevalence of hypertension is 25% in Maharashtra, and a huge variation in the prevalence of hypertension is found across the districts. Dhule, Gadchiroli (with a low HDI rank), Mumbai and Satara (with higher HDI rank) are the districts with the higher (above 30%) prevalence of high blood pressure. The prevalence also significantly varies according to different correlates. The prevalence of high blood pressure is higher among elderly population (40%), among males (28%), in the urban areas (27%) and in the richest wealth quintile (28%). The prevalence is also higher among cigarette smokers (31%), alcohol consumers (30%) and people with obesity (38%) as compared to their counterparts. The results of the multilevel analysis show that the older and obese persons are at four-time higher risk of hypertension. Again, age, sex, marital status, place of residence, wealth status, unhealthy habits (i.e. smoking and alcohol consumption) and BMI are significantly associated with hypertension. The results of VPC statistics show that 14% of hypertension prevalence could be attributed to differences at the community level.

**Conclusion:**

The prevalence of hypertension largely varies in the districts of Maharashtra irrespective of their level of socio-economic development (i.e. HDI rank). The variation in the rate of prevalence of hypertension is higher in the community (PSU) level as compared to the variation in the prevalence rate at the district level. Hypertension is attributable to the modifiable factors like risky lifestyle practices.

## Introduction

Globally, hypertension has been emerging as a serious threat to public health [1]. In 2008, worldwide, approximately 40% of adults aged 25 years and above had been diagnosed with hypertension, raising the total population affected to one billion. Prevalence of hypertension is the highest in the African region (46% of adults aged above 25 years) and lowest in America (35%) [2]. Countries like Spain (40%), Japan (38.3%), Venezuela (39.7%) and Paraguay (35.4%) show a considerably higher rate (>35%) of prevalence of hypertension [3]. Hypertension is a major risk factor that causes approximately 51% of global deaths from stroke and 45% from coronary heart disease [4]. While there is no critical value for blood pressure, the risk of cardiovascular diseases (CVD) and renal failure increases progressively with the level of blood pressure (BP) [4–7]. Different factors are responsible for increasing the prevalence of hypertension in developed and developing countries and are attributable to rapid transition of lifestyle practices in developing countries including India, as well as increased elderly population due to an increase in life expectancy [3, 8–9]. Among the key determinants, age and sex are the important non-modifiable risk factors for hypertension [4, 9]. Besides, the level of education, economic condition, place of residence, religion, community and treatment for hypertension are the potential predictors of diagnosis [10–12]. The other risk factors for hypertension include co-morbidities like obesity, high cholesterol, and diabetes mellitus [2]. Further, a higher level of BMI and alcohol consumption, improper dietary practices and sedentary lifestyle are the major contributing factors to the higher prevalence of hypertension in India [8, 13–16].

During the last few decades, the prevalence of hypertension has been increased drastically in India and in its states. [8, 17]. According to 2008 estimate, hypertension affects more than one out of every five adults in India [18]. Further, there is a dearth of research on hypertension using data, stratified at the community, district and state level. Besides, only a few studies have focused the key factors that influence the prevalence rate of hypertension as a whole as well as on an individual basis. Understanding this collective phenomenon is relevant to both etiologic research and prevention strategies [19]. The present paper aims to investigate the selected factors affecting the hypertension prevalence by using multilevel modeling. It also aims to measure the influence of the combination of selected factors on the current prevalence of hypertension in Maharashtra. Emphasis is given to exploring the true effect of the factors on the prevalence, taking into consideration the effect at different levels. Hence, the study examines and explains how the individual-level characteristics impact hypertension and the difference in the prevalence rate of hypertension at the community and districts level [20].

## Material and methods

### Data and sample

The study uses a secondary data which is obtained from the District Level Household and Facility Survey (DLHS-4), 2012–13. This is a nationally representative sample survey and the data is collected exclusively for each state. The DLHS-4 adopted a multistage stratified sampling procedure. For the purpose of collecting the urban sample, two-stage sampling was used. The primary sampling unit (PSU) was the National Sample Survey Organization (NSSO) urban frame survey (UFS) blocks and second stage sampling unit (SSU) was the household survey. The urban PSU was selected by equal probability sampling without any replacement, and SSU was selected by using circular systematic sampling, according to sampling frame of NSSO 2007–08. In the case of rural area, two-stage sampling was used considering the census village as PSU and household as the SSU. The PSUs were selected using the probability proportional to size (PPS) sampling with replacement and SSUs were selected by systematic circular sampling using sampling frame of census 2001. The data were collected through face to face interviews using different types of schedules such as Household, Women, Village and Facility Questionnaires (see Maharashtra, DLHS-4 report for more information on sample design) [21]. This survey collects information on the key indicators of reproductive and child health care services. But, the last round of DLHS has added information on disability, injury and acute and chronic illness and also the first time this survey uses clinical, anthropometric and biochemical (CAB) test to measure haemoglobin, blood sugar and blood pressure (BP) of all eligible household members aged 18 years and above. Blood pressure of individual was measured using a Ross Max AW150 blood pressure monitor model, the automatic device included separate cut off for measuring BP of individual with small, medium and large arms circumferences. The both systolic and diastolic BP was taken during the survey at approximately ten minutes interval. This study includes a total of 110880 survey participants and among them, 27821 are diagnosed with the problem of hypertension. The distribution of sample according to their demographic and socio-economic characteristics is shown in Table 1.

**Table 1:** Sample distribution by background characteristics of the respondents.

Background Characteristics	Percentage	Number
**Age Group**		
18–29	29.1	32222
30–39	21.3	23602
40–49	17.7	19579
50–59	13.9	15417
60+	18.1	20060
**Sex**		
Male	45.0	49861
Female	55.0	61004
**Marital Status**		
Single	14.4	15981
Married	76.9	85207
Widow/Divorced/Separated	8.7	9622
**Place of Residence**		
Rural	57.3	63583
Urban	42.7	47297
**Education status**		
Illiterate	2.3	1969
Primary	13.6	11701
Secondary	29.4	25402
Higher	54.8	47284
**Religion**		
Hindu	80.0	88711
Muslim	10.7	11897
Buddhist	7.5	8279
Christian	0.5	560
Others	1.3	1399
**Caste**		
Scheduled Caste	19.0	19671
Scheduled Tribe	14.0	14446
Other Backward Class	43.2	44657
Others	23.9	24669
**Wealth Index**		
Poorest	20.6	22789
Poorer	18.8	20802
Middle	18.6	20620
Richer	21.4	23766
Richest	20.7	22903
**Administrative Region**		
Kokan	12.6	14012
Pune	14.5	16087
Nashik	16.6	18376
Amravati	15.6	17293
Nagpur	16.6	18366
Aurangabad	12.2	13512
Nanded	11.9	13234
**Total**	**100.0**	**110880**

### Variables

#### Predictor variables

The principal predictor variables used in the analyses are: *Age* of respondents, which is re-coded in five categories (i.e. 18–29, 30–39, 40–49, 50–59, and 60 and above years); *Gender* (assessed as male and female); *Marital status* (re-coded into three categories i.e. single, married and widow, divorced or separated; *Place of residence* (categorized into rural and urban); *Education level* (categorized as illiterate/uneducated, primary, secondary and higher); *Caste* (categorized as scheduled caste (SC), scheduled tribe (ST), other backward classes (OBC) and general/other category); *Religion* (grouped into five different religions, i.e. Hindu, Muslim, Buddhist, Christians and Others); *Region of residence* (categorized according to seven administrative divisions in Maharashtra, namely Konkan, Pune, Nashik, Amravati, Nagpur, Aurangabad and Nanded) and *Wealth index* with five categories (poorest, poorer, middle, richer and richest). Based on the information received on respondents’ household amenities and assets such as the main source of drinking water, kind of water facility, fuel and source of light, type of household structure, the status of land and household ownership, possession of different assets used in the household etc., an index (i.e. wealth index) was constructed. The answer to each question was assigned a weight (factor score) generated through Principal Component Analysis (PCA). The sample was divided into five equal parts from the poorest to the richest with a cut-off of 20 percent. A higher score indicates a higher economic status. This index was validated while performing “factor analyses” through scree—plot that shows the components as the x-axis and the corresponding eigen values as the y-axis. The internal consistency of the variables used in creating the wealth index was checked and validated (α = 0.8281).

Other important predictor variables are:

*Body Mass Index (BMI)*: Body mass index is calculated using the formula: weight (kilogram) /height^2^ (meter^2^), and classified according to the WHO classification. The BMI scores are grouped into three categories, i.e. BMI<18.5 or thin, BMI = 18.5–24.9 or Normal, and BMI ≥25 or overweight/obese.
*Diabetes*: The individual blood glucose is measured using portable glucometer namely SD free. Here, we have considered individual as diabetic when his/her blood sugar level is ≥140 milligram/deciliter.

The alcohol consumer, tobacco chewer and smoker are defined as those who ever had consumed alcohol, ever had used any type of tobacco and ever had smoked respectively in their lifetime. Hence, the variables *tobacco use*, *smoking*, and *alcohol consumption* are grouped into three categories, i.e. current user, ex-user/former user, and not user.

#### Outcome variables

The key outcome variable, considered in the study is *high blood pressure*. Blood pressure (BP) measurements of ≥140 millimetre of mercury (mmHg) for systolic blood pressure (SBP) and/or ≥90 mmHg for diastolic blood pressure (DBP) are considered as high blood pressure. Hypertension is defined as the presence of persistently elevated BP or history of treatment with anti-hypertensive agents [3, 22].

### Statistical analysis

We have calculated the prevalence of hypertension in association with other factors presented in the form of a percentage. The association between the categorical variables and the prevalence of hypertension is tested using chi-square test at 5% significance level. Multilevel logistic regression analysis is performed to assess independent risk factors for hypertension. The *p*-values of <0.05 are considered statistically significant [22].

#### Multilevel Logistic Regression (MLR) model

Due to the hierarchal structure of the dataset, where individuals (level 1), are nested within the community (PSU), who are in turn nested within districts, a multilevel logistic regression model is applied to this study. Thus, a multilevel model with three levels is used to identify the determinants of hypertension at the individual, community and district level (i.e. fixed part model). In addition, the random intercept model is used to measure the random effect or the clustering of participants at community and district level. This study has fitted three separate models for the analysis of the prevalence of hypertension and the effects of its determinants at different levels.

Model 1 (or empty model) contains no exposure variables and only focuses on decomposing the total variance into the community (PSU) and the district components. Model 2 contains only respondents’ achieved and ascribed characteristics, such as age, sex, education, wealth status, religion, caste, residence, and region. The last model (model 3) includes respondents’ health and behavioural characteristics, such as BMI, blood sugar level, use of tobacco, smoking and alcohol consumption. The result of fixed effect parts of the models is presented in the form of odds ratio (ORs) with 95% confidence intervals (CIs).

This type of analysis (i.e. MLR) allows us to partition the variation in the outcome variable (*i*.*e*. Blood pressure above ≥140 millimeter of mercury (mmHg) for systolic blood pressure (SBP) and/or ≥90 mmHg for diastolic blood pressure or DBP) measured at individual level and the variation is attributable to differences among individuals, communities and district level. The multilevel logistic model, considering clustering of outcome variable can be expressed as follows

$$logit(p_{ijk})=\log(\frac{p_{ijk}}{1-p_{ijk}})=\alpha +\beta^{\prime }x_{ijk}+u_{jk}+v_{k}$$

Where $\log(\frac{p_{ijk}}{1-p_{ijk}})$ is the logit function in which *p*_*ijk*_ is the probability of person ‘i’ is the community ‘j’ is the district, ‘k’ having hypertension. The ‘α’ is the constant, and *μ*_*jk*_ and *υ*_*k*_ are the area level residual explained at community and district level.

The result of random effect part of the model is presented as the variance partition coefficient (VPC) which is able to measure both cluster and individual level variance. The Median Odds Ratio (MOR) is calculated only to measure cluster level variance and they are not equivalent, and therefore they are used to measure different aspects [19].

Variance Partition Coefficient (VPC): We have used latent variable approach to estimate variance partition coefficient. This approach was first time used by Snijders and Bosker in 1999 [23]. This approach is useful in the analysis of binary response variables, where we assume a standard logistic distribution for binary outcome variable. The VPC is used to measure the proportion of total variance in the outcome variable, which is attributable to community and district level. The VPC can be expressed as:

$${VPC}_{c}=\frac{\sigma_{c}^{2}+\sigma_{d}^{2}}{\sigma_{c}^{2}+\sigma_{d}^{2}+\frac{\pi^{2}}{3}}$$

$${VPC}_{d}=\frac{\sigma_{d}^{2}}{\sigma_{c}^{2}+\sigma_{d}^{2}+\frac{\pi^{2}}{3}}$$

Where, $\sigma_{c}^{2}$ is the community level variance, and $\sigma_{d}^{2}$ shows district-level variance. The variance for the standard logistic distribution is $\frac{\pi^{2}}{3}\approx 3.29$.

Median Odds Ratio (MOR): This is a widely used indicator, where we can able to translate variance into more widely used indicator *i*.*e*. odds ratio. It is useful to measure only cluster level variance, where VPC and MOR are not equivalent; therefore they are used to measure different aspects. The MOR is defined as the median value of the odds ratio between the area of high risk and the area of low risk, while randomly picking out an area between these two areas. In this study, MOR shows that individual probability of having hypertension is determined at community (i.e. PSU) and district level, where variances of each level are converted into MOR. The MOR equal to one indicates that there would be no geographical difference and MOR greater than one indicates that geographical variables play an important role in the odds of reporting of hypertension. The MOR can be expressed as:

$$MOR=exp[\sqrt{\left(2*V_{A}\right)}*0.6745]$$

$$\approx exp(0.95*\sqrt{V_{A}})$$

Where V_A_ is the area level variance and 0.6745 is the 75^th^ centile of the cumulative distribution function of the normal distribution with mean 0 and variance 1.

All the statistical analyses are performed in STATA 13 and MLwiN 2.28 software. However, ArcGIS 10 is used in creating the map.

## Results

### Prevalence of hypertension in Maharashtra

In Maharashtra, the prevalence of hypertension is found 25.1%. Besides, a huge variation in the prevalence of hypertension is observed across the districts (S1 Table).

**Fig 1:**
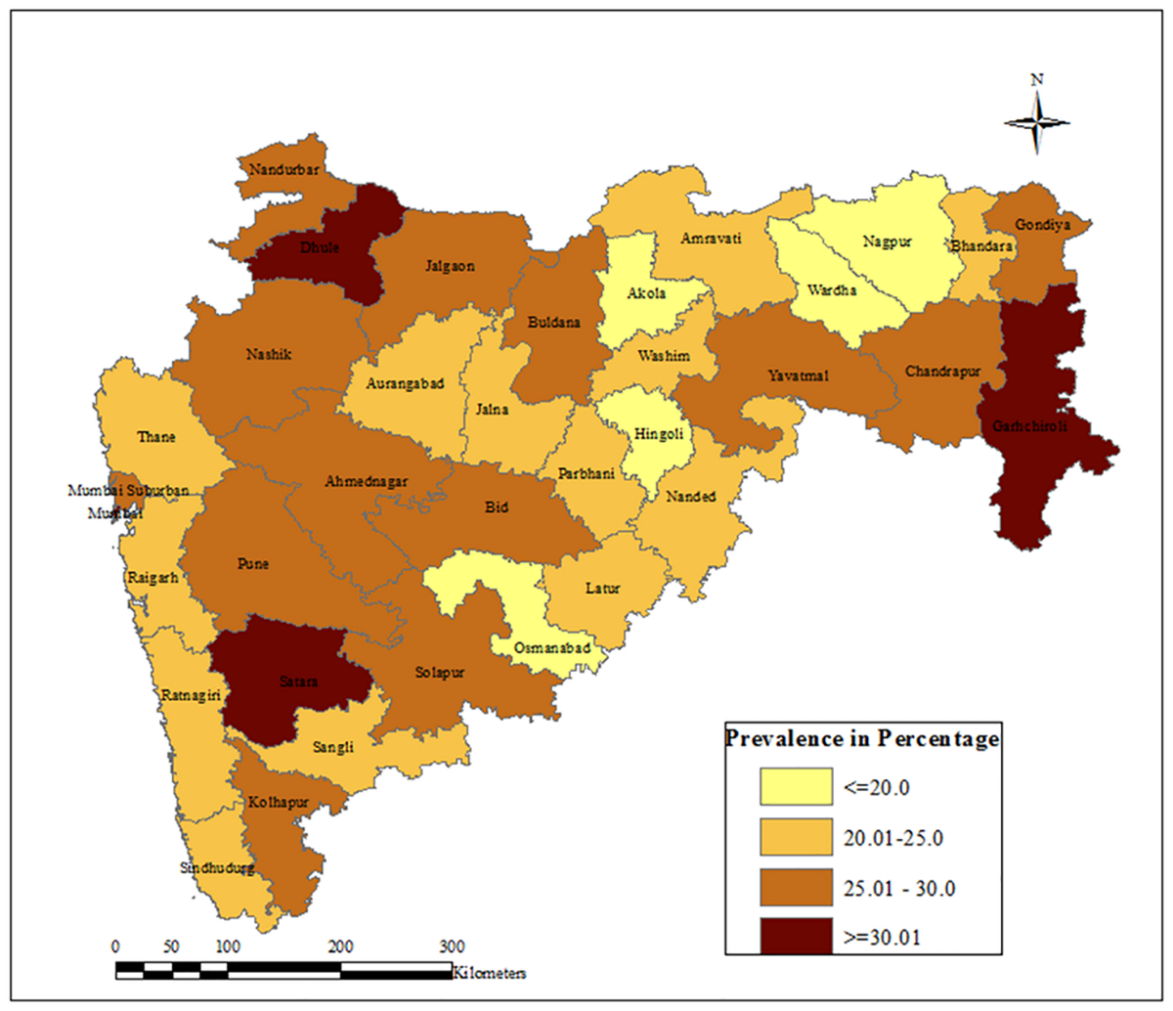
Prevalence of hypertension in Maharashtra, 2012–13.

At the district level, the prevalence rate of hypertension is varied between 15% in Hingoli and 36% in Mumbai. Satara, Dhule, Gadchiroli and Mumbai are the districts with more than 30% prevalence of high BP, whereas Hingoli, Nagpur, Osmanabad, Wardha and Akola have a prevalence rate of less than twenty percent. There are districts like Nandurbar, Jalgaon, Buldana, Gondiya, Chandrapur, Yavatmal, Mumbai Suburban, Pune, Ahmednagar, Bid and Solapur which show a higher prevalence of hypertension than the state average (Fig 1).

### Prevalence of hypertension associated with different socioeconomic factors

Table 2 shows the prevalence of hypertension in Maharashtra according to the socioeconomic characteristics of the respondents. About one-fourth (25%) of the respondents are at the risk of hypertension. The study shows that the prevalence of hypertension is positively associated with the age of the respondents and the results are found statistically significant (χ^2^ = p<0.001).

**Table 2:** Prevalence of hypertension by socioeconomic characteristics of the respondents, Maharashtra, 2012–13.

Socio-economic characteristics	Blood Pressure (>140 systolic & >90 diastolic) (%)	95% CI (%)	Total (N)
**Age Group (years)**			
18–29	13.7	13.0–14.4	32222
30–39	20.1	19.4–20.9	23602
40–49	27.1	26.2–28.0	19579
50–59	33.7	32.7–34.7	15417
60 & above	40.7	39.8–41.6	20060
**Sex**			
Male	27.9	27.2–28.6	49861
Female	22.8	22.2–23.4	61004
**Marital Status**			
Single	15.9	15.0–16.8	15981
Currently married	25.5	24.8–26.1	85207
Widow/divorced/separated	37.0	35.9–38.1	9622
**Place of Residence**			
Rural	23.9	23.1–24.7	63583
Urban	26.7	25.7–27.7	47297
**Education level**			
Illiterate/uneducated	26.4	24.1–28.8	1969
Primary	28.6	27.6–29.7	11701
Secondary	23.1	22.3–23.9	25402
Higher	22.2	21.4–23.0	47284
**Religion**			
Hindu	24.8	24.2–25.5	88711
Muslim	27.8	26.1–29.5	11897
Buddhist	23.1	21.9–24.4	8279
Christian	29.7	23.1–37.3	560
Others	28.7	25.5–32.0	1399
**Caste**			
Scheduled Castes (SCs)	23.8	22.9–24.7	19671
Scheduled Tribes (STs)	26.2	24.6–27.8	14446
Other Backward Classes (OBCs)	25.3	24.5–26.1	44657
Others	25.0	24.0–26.1	24669
**Wealth Index**			
Poorest	24.2	23.1–25.3	22789
Poorer	22.8	21.9–23.7	20802
Middle	24.0	23.2–24.9	20620
Richer	25.7	24.8–26.6	23766
Richest	28.4	27.2–29.7	22903
**Administrative Regions**			
Kokan	24.8	23.2–26.5	14012
Pune	27.5	26.0–29.0	16087
Nashik	28.5	26.8–30.3	18376
Amravati	24.0	22.6–25.3	17293
Nagpur	25.3	23.3–27.3	18366
Aurangabad	22.9	21.7–24.1	13512
Nanded	21.1	19.8–22.5	13234

**Fig 2:**
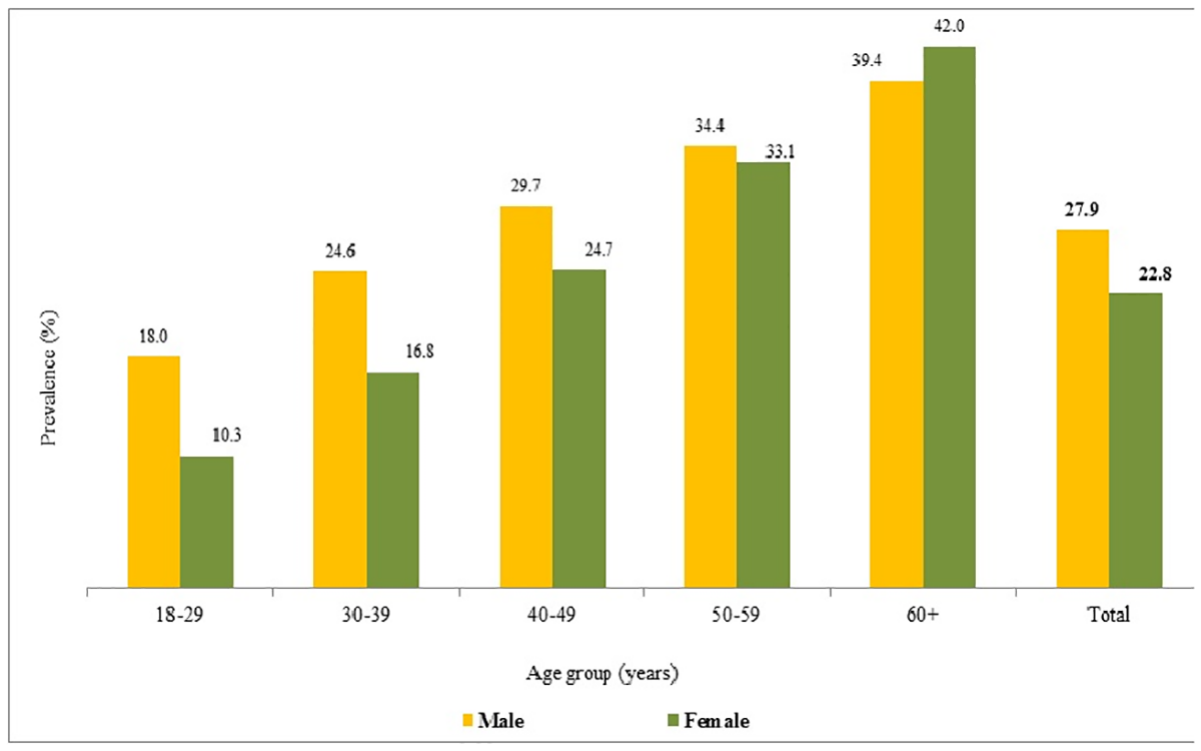
Prevalence of hypertension by age group and sex, Maharashtra, 2012–13.

Fig 2 shows that the prevalence of hypertension in males (28%) is significantly higher as compared to the prevalence in females (23%). But, in the age group of sixty years and above, the prevalence of hypertension is found higher among females (42%) than among males (39%).

**Fig 3:**
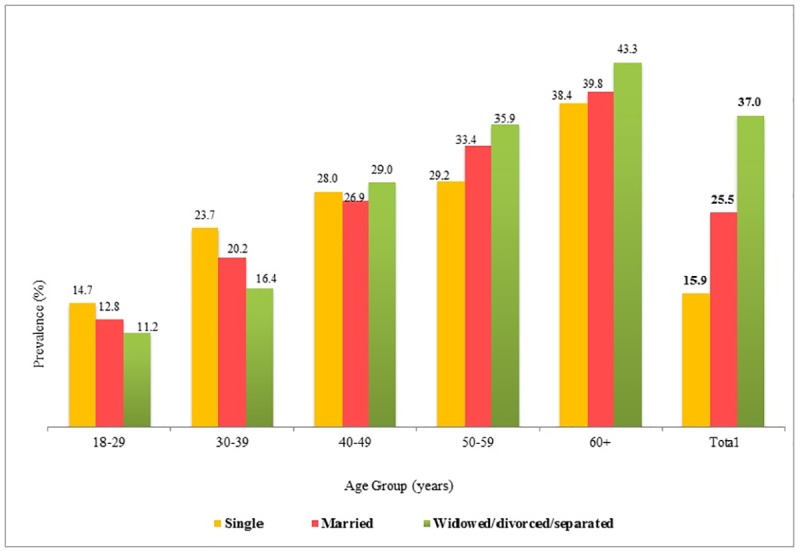
Prevalence of hypertension by age group and marital status, Maharashtra, 2012–13.

We have observed a higher prevalence of hypertension among widow, divorced and separated (37%, 95% CI: 35.9–38.1), compared to single and married. In contrast, when it is compared to the former, a higher prevalence of hypertension is found among the single and married at the younger age (i.e. age between 18 and 39 years). The prevalence increases after the age of forty years among widowed, divorced and separated (Fig 3). We have also found an urban-rural difference in the prevalence of hypertension, which is 27% (95% CI: 25.5–27.7) in the urban areas and 24% (95% CI: 23.1–24.7) in the rural areas. No clear pattern of prevalence of hypertension by education is seen, although respondents who are illiterate and are with primary education have a higher risk of hypertension as compared to those who have higher education.

The prevalence of hypertension is found higher in Christian and Muslim religion. Again, respondents belong to Scheduled Tribe (ST) are found to experience a higher level of BP (i.e. 26%, 95% CI: 24.6–27.8) than the respondents from other castes. We have observed a higher prevalence of hypertension (i.e. 28%, 95% CI: 27.2–29.7) among the respondents belonging to the richest wealth quintile as compared to the other respondents. Further, across the seven administrative divisions, variation in the prevalence of hypertension is also observed. Nashik (28.5%, 95% CI: 26.8–30.3) and Pune (27.5%, 95% CI: 26.0–29.0) regions have shown a higher prevalence of hypertension.

### Prevalence of hypertension associated with health and lifestyle factors

Table 3 shows the prevalence of hypertension according to health and lifestyle practices of the respondents. The people with obesity (BMI ≥25) have a high prevalence of hypertension (38%, 95% CI: 36.9–38.8) as compared to the normal (BMI 19.0–24.9), and thin (BMI<18) people. The prevalence of hypertension is significantly higher (37%, 95% CI: 36.2–38.2) among the respondents with a higher level of blood sugar (>140mg/dl) as compared to those who have a low level (<140mg/dl) of blood sugar.

**Table 3:** Prevalence of hypertension by health and life style practices of the respondents, Maharashtra, 2012–13.

Health and lifestyle practices	Blood Pressure (>140 systolic & >90 diastolic) (%)	95% CIs (%)	Total (N)
***Health Indicators***			
**BMI Level**			
<18.5	14.8	14.1–15.5	17031
18.5–24.9	26.1	25.1–27.1	45955
25 & above	37.8	36.9–38.8	16432
**Diabetes**			
≤140 mg/dl	23.3	22.6–23.9	95948
>140 mg/dl	37.2	36.2–38.2	14326
***Lifestyle practices***			
**Tobacco Use**			
No	24.0	23.3–24.7	77879
Current user	29.0	28.1–30.0	22707
Ex-user	30.2	27.6–32.8	1619
**Smoking**			
No	24.8	24.2–25.2	96291
Current smoker	30.2	28.4–32.1	3742
Ex-smoker	31.7	29.5–34.0	2167
**Alcohol consume**			
No	24.7	24.1–25.4	93010
Current consumer	28.7	27.3–30.2	6543
Ex-consumer	32.1	30.1–34.2	2651
**Total**	**25.1**	**24.5–25.7**	**110880**

**Note**: χ^2^ test significant at <0.001 level.

However, lifestyle practices are significantly associated with the prevalence of hypertension. The prevalence rate of hypertension is higher among ex-tobacco users (30%, 95% CI: 27.6–32.8) and current users (29%, 95% CI: 28.1–30.0) as compared to non-tobacco users. Similarly, among ex-smokers (32%, 95% CI: 29.5–34.0) and current smokers (30%, 95% CI: 28.4–32.1), and among ex-consumers (32%, 95% CI: 30.1–34.2) and current consumers (29%, 95% CI: 27.3–30.2) of alcohol, the prevalence rate of hypertension is found higher as compared to the prevalence rate among their counterparts.

### Hypertension associated with different risk factors at different level: Results of multilevel analysis

Table 4 represents three different models which use different exposure variables to show their association with the prevalence of hypertension. Model 1 (or empty model) contains no exposure variables, and the higher values of VPC and MOR at the village or community level (i.e. primary sampling unit or PSU) show a higher variation in the prevalence rate of hypertension.

**Table 4:** Results of multilevel analysis of prevalence of hypertension, Maharashtra, 2012–13.

Background Variable	Model 1^a^		Model 2^b^			Model 3^c^		
	Odds (p values) (SE)	95% CIs	Odds (p values)	SE	95% CIs	Odds (p values)	SE	95% CIs
**Age group (years)**								
18–29^®^			1.00			1.00		
30–39			1.74***	0.05	1.64–1.85	1.63***	0.06	1.51–1.76
40–49			2.68***	0.09	2.51–2.85	2.43***	0.10	2.24–2.64
50–59			3.69***	0.13	3.44–3.96	3.37***	0.15	3.08–3.69
60 & above			4.70***	0.18	4.37–5.06	4.55***	0.22	4.14–4.99
**Sex**								
Male^®^			1.00			1.00		
Female			0.67***	0.01	0.64–0.69	0.69***	0.02	0.66–0.73
**Marital status**								
Single^®^			1.00			1.00		
Married			0.96	0.03	0.90–1.03	0.89**	0.04	0.82–0.97
Widow/Divorced/Separated			1.20**	0.06	1.08–1.33	1.12	0.07	0.98–1.27
**Place of residence**								
Rural^®^			1.00	0.00		1.00		
Urban			1.12**	0.04	1.03–1.21	1.04	0.05	0.95–1.14
**Education level**								
Illiterate/uneducated^®^			1.00			1.00		
Primary			1.21**	0.08	1.06–1.38	1.08	0.10	0.91–1.29
Secondary			1.18*	0.08	1.04–1.35	1.03	0.09	0.87–1.23
Higher			1.13	0.08	0.99–1.29	1.00	0.09	0.84–1.19
**Religions**								
Hindu^®^			1.00			1.00		
Muslim			1.19***	0.05	1.10–1.29	1.12*	0.06	1.01–1.23
Buddhist			0.98	0.04	0.90–1.08	1.04	0.06	0.93–1.16
Christian			1.19	0.16	0.92–1.54	1.16	0.19	0.84–1.60
Others			1.01	0.09	0.86–1.20	0.95	0.10	0.77–1.17
**Castes**								
Scheduled Castes (SCs)^®^			1.00			1.00		
Scheduled Tribes (STs)			1.08	0.04	1.00–1.17	1.09	0.06	0.98–1.20
Other Backward Classes (OBCs)			0.94*	0.03	0.88–1.00	0.95	0.04	0.88–1.03
Others			0.91**	0.03	0.85–0.97	0.91*	0.04	0.83–0.99
**Wealth Index (WI)**								
Poorest^®^			1.00			1.00		
Poorer			1.05	0.04	0.98–1.13	1.03	0.05	0.94–1.12
Middle			1.18***	0.04	1.10–1.27	1.08	0.05	0.99–1.18
Richer			1.22***	0.05	1.14–1.32	1.05	0.05	0.96–1.16
Richest			1.34***	0.05	1.24–1.45	1.06	0.05	0.96–1.18
**Regions**								
Konkan ^®^			1.00			1.00		
Pune			1.22	0.15	0.97–1.54	1.20	0.16	0.92–1.56
Nashik			1.24	0.15	0.98–1.57	1.39*	0.18	1.07–1.80
Amravati			1.03	0.12	0.81–1.30	1.04	0.14	0.80–1.35
Nagpur			0.99	0.11	0.79–1.24	1.05	0.13	0.81–1.35
Aurangabad			0.97	0.12	0.75–1.24	1.13	0.16	0.85–1.51
Nanded			0.87	0.11	0.67–1.11	0.91	0.13	0.69–1.22
**Body Mass Index (BMI)**								
<18.5^®^						1.00		
18.5–24.9						2.02***	0.07	1.88–2.17
25 & above						3.71***	0.15	3.42–4.02
**Diabetes (mg/dl)**								
≤140 ^®^						1.00		
>140						1.35***	0.04	1.26–1.44
**Tobacco use**								
No^®^						1.00		
Current User						1.01	0.03	0.95–1.08
Ex-user						1.13	0.10	0.94–1.35
**Smoking**								
No^®^						1.00		
Current smoker						1.11	0.07	0.98–1.26
Ex-smoker						1.05	0.09	0.89–1.23
**Alcohol consumption**								
No^®^						1.00		
Current consumer						1.04	0.05	0.95–1.15
Ex-consumer						0.90	0.07	0.78–1.04
**Random Effect Part**								
**Variance (SE)**^#^								
District	0.044(0.013)	0.02–0.07	0.026	0.01	0.01–0.04	0.030	0.01	0.01–0.06
PSU	0.394(0.016)	0.36–0.43	0.464	0.02	0.42–0.50	0.510	0.03	0.46–0.56
**VPC(%)**^¶^								
Level 3 (Dist)	**1.2%**		**0.7%**			**0.9%**		
Level 2 (PSU)	**11.7%**		**13.0%**			**14.2%**		
**Median Odds Ratio (MOR)**								
MOR_Dist_	**1.21**		**1.18**			**1.18**		
MOR_PSU_	**1.86**		**1.94**			**2.01**		

Note:

^**a**^Model 1 (or empty model) contains no exposure variables, and only focuses the decomposing of total variance into community (PSU) and district components;

^b^Model 2 contains only respondents’ achieved and ascribed characteristics such as age, sex, education, wealth status, religion, caste, residence, and region;

^c^Model 3 includes respondents’ health and behavioral characteristics such as BMI, blood sugar level, use of tobacco, smoking and alcohol consumption;

^®^Reference category;

Significance level:

***p<0.001,

**p<0.01,

*p<0.05;

^#^ Variance expressed in standard error;

^**¶**^Variance partition coefficients (VPC).

A separate model (model 2), which has adjusted age, sex, marital status, place of residence, education level, religions, castes, and regions, shows a statistically significant association between the stated socio-economic variables and hypertension. The age of respondents is positively associated with hypertension and an odds ratio (OR) 4.7 (p<0.001, 95% CI = 4.37–5.06) for older persons (60 years and above) indicates that they are four times more likely at the risk of hypertension than younger persons (18–29 years). It is found that males are also at the higher risk of hypertension than females. The positive association between marital status and hypertension shows that widows, divorced or separated are significantly more likely (OR = 1.20, p<0.001) to be hypertensive as compared to those who are never married or single. Respondents from urban areas (OR = 1.12, p<0.001), Muslim religion (OR = 1.19, p<0.001) and richer wealth quintiles (OR = 1.18–1.34, p<0.001) are significantly more likely to be at the higher risk of hypertension as compared to their counterparts. In contrast, respondents from the other backward classes (OBCs) (OR = 0.94, p<0.05) and general class (OR = 0.91, p<0.01) are significantly less likely at the risk of hypertension as compared to respondents from scheduled castes community.

A third model (model 3), adjusted for behavioral characteristics along with the socio-economic characteristics, shows that the respondents from higher age i.e. 60 years and above (OR = 4.55, p<0.001), Muslim religion (OR = 1.12, p<0.05), and with a higher level of BMI (i.e. 25 and above) (OR = 3.71, p<0.001) and a higher level of blood sugar (i.e.>140 mg/dl) (OR = 1.35, p<0.001) are significantly more likely to experience hypertension as compared to their respective reference population. Respondents from the Nashik region are also more likely (OR = 1.39, p<0.05) to experience hypertension as compared to those from Konkan region.

Our analysis explores the variation in the prevalence of hypertension between district and community (PSU) level in Maharashtra. In comparison to model 1, higher variation in the prevalence of hypertension is observed in Model 2, i.e. variance partition coefficient (VPC) at the district and community level, which contributes 0.7% and 13% respectively to the total variation in the prevalence of hypertension. Further, model 3 shows an increase in the variation in the prevalence of hypertension at the community level (i.e. 14.2%). Similarly, median odds ratio (MOR) from the model 1 shows a geographical variation in the prevalence of hypertension in Maharashtra. Overall, the variation in the prevalence of hypertension is more (MOR = 1.86–2.01) at the community level (i.e. PSU), although the variation at the district level is less (MOR = 1.18–1.21).

## Discussion

Maharashtra ranks among the top five ranked states in India on the Human Development Index (HDI). The present study focuses the scenario of hypertension in Maharashtra and its districts. The study has found a huge variation in the rate of prevalence of hypertension in the districts of Maharashtra. The prevalence of hypertension is found the lowest (15%) in Hingoli (the district with low HDI rank), and the highest (36%) in Mumbai (very high HDI ranked district). Besides, regional variation is observed and hypertension is found more prevalent in Pune and Nashik (regions sharing a higher proportion of Net State Domestic Product).

Among the individual level non-modifiable factors, age, sex and marital status are found to be significantly associated with hypertension. Similar to the previous studies, older participants, males, widowed, divorced and separated, Christians and Muslims, scheduled tribes, low educated and wealthier respondents are more likely to be hypertensive[24–26].

Further, the prevalence rate of hypertension is found higher in urban areas than in rural areas [27, 28]. In this study, we have also found that a higher level of BMI (25 and above) and level of blood glucose (>140 mg/dl) are also the significant predictors of hypertension and are positively associated with the prevalence of hypertension [27, 29]. Other lifestyle factors which are modifiable, such as consumption of tobacco and alcohol and habit of smoking also play an important role in increasing the risk of hypertension [25, 30, 31]. Despite awareness of the risk factors of hypertension, even among the participants with a higher level of education and economic status, prevalence of hypertension may be high due to their obesity, physical inactivity and the place of residence i.e. urban areas. Higher reporting of the problem of high blood pressure level among wealthier and educated respondents is also considered as an important factor for the higher level of prevalence of hypertension in this group [18, 27].

In the present study, the purpose of using multilevel model is to assess the amount of variability in the prevalence of hypertension due to the effects of the different combination of factors and due to the effect of each level [19, 32]. Hence, the smaller value of VPC (1%) indicates that variation in the rate of prevalence of hypertension at the district level is low that may be due to the influence of other factors (i.e. socio-economic characteristics, health and lifestyle practices). On the contrary, at the community level a higher value of VPC (13% to 14%) shows a higher rate of variation in the prevalence of hypertension and can be attributed to the effects of background factors [24].

Hypertension is the third most important risk factor for burden of diseases in South Asia [25]. Overall, the current prevalence of hypertension in Maharashtra is 25% which is below the world average. The rate is consistent with many Asian countries like Bangladesh (24%), China (24%) and Vietnam (25%), but is inconsistent with the western countries like United States (20.3%) and Canada (21.4%) [2,3,10]. According to a study, 20.6% of Indian men and 20.9% of Indian women were suffering from hypertension in 2005 [3]. The reported prevalence of hypertension was 37% among 30–64 age group in 1998 and 55% among 40–60 age group in 2000 in India, though the rate varies from rural to urban and across the states [5, 9]. Action on the improvement of lifestyle and its management to reduce the risk of hypertension is limited in India. Further, inappropriate food habits and lifestyle practices are also responsible for the higher prevalence of hypertension [18, 25, 31]. Proper blood pressure control measure is needed to avoid the risk of hypertension [33]. Hence, the control of the identified risk factors (such as the use of tobacco and consumption of alcohol) across the economic status can improve the hypertension level of the population in the state of Maharashtra.

## Conclusion

Hypertension is more common in urban areas than in rural areas in India. The multilevel analysis has shown that the variation in the rate of prevalence of hypertension is higher at the community (PSU) level as compared to the variation in the prevalence rate at the district level in Maharashtra. The study also explores the high prevalence rate of hypertension in Pune, Nashik and Amravati regions. The study emphasises the need of an additional focus and strategy to control hypertension among the disadvantaged population (like uneducated and low educated, widowed, divorced and separated, scheduled caste and scheduled tribe, and the religious minority communities like Christians and Muslims) among whom hypertension is more prevalent. The results accentuate the existing potential for preventive strategies, focusing the community and direct for both medical and lifestyle-related factors such as medical counselling, appropriate dietary practices and societal attitudes toward healthy lifestyles. This will help to achieve an optimum BMI level and a low blood glucose level. Moreover, proper guidelines and promotion of healthy practices to adhere a healthy lifestyle are required. Further, changes in unhealthy behaviour and practices at the individual level are also desired. At the community and district level, there is an urgent need for appropriate intervention strategies for the prevention and control of hypertension by providing information, early diagnosis, and treatment.

## Strength and limitations

Very few multilevel analysis have been carried out in India using hierarchical data on hypertension. This is the first ever study, not only in Maharashtra but in India, to present recent information on hypertension (affected by a wide range of individual, household and community level characteristics) using a state-level survey data. Further, the present study indicates a variation in the prevalence rate of hypertension at the community and district level and the risk factors of hypertension among the survey participants backed by scientific evidence. For the first time haemoglobin, blood sugar and blood pressure of the survey participants were tested in a large-scale demographic survey in India using clinical, anthropometric and biochemical (CAB) examination, and these measurements are directly used in the study. The prevalence of hypertension in this study is estimated only for the survey participants. The details of the measurement of blood pressure and blood sugar can be found in the survey report. Hence, the estimated prevalence of hypertension in this study, based on a large-scale sample survey data, may differ to an extent from the actual prevalence of hypertension in the state, although, the estimated prevalence of hypertension in this study is consistent with the prevalence estimated by the other surveys [34].

## Supporting information

S1 Table (DOCX)
