# Supplementary material for: Prevalence and correlates of hypertension in Maharashtra, India: A multilevel analysis
Source: PLoS One. 2018 Feb 5;13(2):e0191948. doi: 10.1371/journal.pone.0191948 (PMC5798824; doi:10.1371/journal.pone.0191948)
Supplement: S1 Table — (DOCX) [file pone.0191948.s001.docx]

| **S1Table. Prevalence of Hypertension by Districts, Maharashtra, India, 2012-13** | | | |
| --- | --- | --- | --- |
| **Districts** | **Blood pressure**  **(>140 systolic & >90 diastolic) (%)** | **Having high blood pressure**  **(n)** | **Total**  **(N)** |
| Nandurbar | 27.7 | 850 | 3088 |
| Dhule | 32.0 | 1458 | 4550 |
| Jalgaon | 27.6 | 1002 | 3626 |
| Buldana | 28.4 | 1126 | 3992 |
| Akola | 19.1 | 731 | 3803 |
| Washim | 21.5 | 647 | 3027 |
| Amravati | 23.5 | 837 | 3560 |
| Wardha | 19.1 | 683 | 3596 |
| Nagpur | 17.9 | 441 | 2471 |
| Bhandara | 24.5 | 591 | 2433 |
| Gondiya | 25.8 | 698 | 2718 |
| Gadchiroli | 32.7 | 1050 | 3226 |
| Chandrapur | 29.5 | 1147 | 3922 |
| Yavatmal | 27.3 | 804 | 2911 |
| Nanded | 22.0 | 796 | 3627 |
| Hingoli | 15.3 | 361 | 2353 |
| Parbhani | 22.5 | 775 | 3463 |
| Jalna | 23.9 | 1099 | 4593 |
| Aurangabad | 23.7 | 806 | 3347 |
| Nashik | 25.7 | 848 | 3352 |
| Thane | 25.1 | 494 | 1969 |
| Mumbai (Suburban) | 26.1 | 556 | 2150 |
| Mumbai | 36.1 | 547 | 1521 |
| Raigarh | 22.5 | 866 | 3782 |
| Pune | 29.3 | 914 | 3103 |
| Ahmadnagar | 28.5 | 1074 | 3760 |
| Bid | 25.4 | 643 | 2523 |
| Latur | 22.7 | 878 | 3791 |
| Osmanabad | 18.3 | 565 | 3049 |
| Solapur | 28.7 | 916 | 3191 |
| Satara | 30.7 | 771 | 2522 |
| Ratnagiri | 21.5 | 453 | 2096 |
| Sindhudurg | 22.8 | 581 | 2494 |
| Kolhapur | 26.1 | 876 | 3392 |
| Sangli | 24.3 | 937 | 3879 |
| **Total** | **25.1** | **27821** | **110880** |
